# Construction and interpretation of tobacco leaf position discrimination model based on interpretable machine learning

**DOI:** 10.3389/fpls.2025.1619380

**Published:** 2025-07-25

**Authors:** Ranran Kou, Cong Wang, Jinxia Liu, Ran Wan, Zhe Jin, Le Zhao, Youjie Liu, Junwei Guo, Feng Li, Hongbo Wang, Song Yang, Cong Nie

**Affiliations:** ^1^ Key Laboratory of Tobacco Chemistry, Zhengzhou Tobacco Research Institute of China National Tobacco Corporation (CNTC), Zhengzhou, China; ^2^ Technology Center, China Tobacco Jilin Industrial Co., Ltd., Changchun, China

**Keywords:** tobacco leaf chemical components, position discrimination, analysis of crop quality, model interpretation, SHapley Additive exPlanations (SHAP)

## Abstract

Tobacco leaf position is closely associated with its quality whose material basis is the chemical components of tobacco leaf. In recent years, near-infrared (NIR) spectroscopy combined with algorithmic models has emerged as a popular method for identifying the tobacco leaf position. However, when applied to leaf position discrimination, these models often rely on principal components derived from dimensionality-reduced spectral signals, resulting in limited interpretability and difficulty in identifying key chemical components. Chemical composition data combined with algorithmic models can also be used to discriminate tobacco leaf positions. However, the acquisition of chemical components relies on traditional instrumental analytical methods. As a result, the acquisition of chemical composition data is time-consuming and labor-intensive, involving only a limited number of compounds. The study proposes a novel approach that integrates machine learning with advanced interpretability techniques for both tobacco leaf position discrimination and analysis. Based on the 70 tobacco leaf chemical components obtained using near-infrared rapid analysis technology, tobacco leaf position discrimination models were built using Support Vector Machine (SVM), Back Propagation Neural Network (BPNN), and Random Forest (RF). Particle swarm optimization (PSO) was used to optimize parameters of each model. Chemical components were analyzed for statistical significance across leaf positions, and their influence on model predictions was interpreted using SHapley Additive exPlanations (SHAP). The experimental results showed that among all models, the SVM- hybrid kernel demonstrated the most robust and accurate performance, achieving discrimination accuracies of 98.17% and 96.33% on the training and test sets, respectively. SHAP analysis provided a clear ranking of feature importance and revealed the positive and negative contributions of individual chemical components. The proposed method can be useful for position traceability and chemical feature analysis of various crops.

## 1 Introduction

The quality of crops is closely related to the growing positions (Guleria et al., 2007), and the light temperature and nutrient distribution of plants in different positions are different, thus affecting the yield and quality. Tobacco is an important economic crop for many countries and plays an important role in promoting farmers’ income and government tax revenue (Drope et al., 2022; Zhao, 2022). In the tobacco industry, tobacco leaves are generally divided into upper, middle and lower positions for evaluation. It is generally believed that the middle leaves are of better quality than the upper or lower leaves, so the price of the middle leaves is higher than the upper or lower leaves in the tobacco market. Therefore, the market sometimes deliberately mixes upper and lower tobacco leaves into middle tobacco leaves to pursue higher profits, or involuntarily mislabels and mixes tobacco leaves in different positions (Ni et al., 2009), so it is of great significance to accurately identify tobacco leaf positions. In addition, the material basis of tobacco quality is the chemical components of tobacco leaves. It is also meaningful to clarify the chemical characteristics of different positions of tobacco leaves and take corresponding measures to improve quality.

In recent years, machine learning (ML) has made significant advances in areas such as computer vision (Krizhevsky et al., 2017; Rawat and Wang, 2017), machine translation (Zhang and Zong, 2015), fault detection (Theissler, 2017), and predictive maintenance (Theissler et al., 2021). Near-infrared spectroscopy has the advantages of simple sample pretreatment, non-destructive sample, fast analysis speed, good repeatability and reproducibility, and has been widely used in recent years (Wang et al., 2018a; Richter et al., 2019; Wu et al., 2020; Xiao et al., 2020). The combination of near-infrared spectroscopy and machine learning has been extensively employed for identifying tobacco origin and leaf position. Wang and Yang (2023) proposed a generalized learning system of Takagi-Sugeno (TS) fuzzy subsystem based on near-infrared spectroscopy for rapid identification of tobacco origin. Xiang et al. (2020) conducted identification of the geographical origin and grade of flue-cured tobacco based on near-infrared spectroscopy. Based on NIR spectral data of tobacco leaves, Bin et al. (2016) proposed an improved random forest method to classify tobacco grades. He et al. (2024) based on near infrared spectroscopy, combined with linear discriminant analysis (LDA) and random subspace method (RSM), built an RSM-LDA integrated learning model for the identification of tobacco positions. Yu et al. (2011) used the mathematical model of similarity analysis based on SIMCA algorithm to conduct similarity analysis of near infrared spectra of tobacco leaves in different positions. Yang et al. (2014) took the near-infrared spectroscopy of tobacco samples as the test object and established the tobacco leaf position projection analysis model based on principal component and Fisher criterion (PPF). However, when near-infrared spectroscopy is combined with algorithmic models to characterize tobacco positions, these models often rely on principal components derived from dimensionality-reduced spectral signals, resulting in limited interpretability and difficulty in identifying key chemical components.

Chemical composition data combined with algorithmic models can also be used to discriminate tobacco leaf positions. Sha et al. (2019) developed a support vector machine (SVM) classification model to distinguish between middle and upper tobacco leaves based on six chemical components: scopoletin, rutin, malic acid, citric acid, fructose, and sucrose. Xie et al. (2008) used a discriminant method based on Mahalanobis distance to identify tobacco leaf positions using chemical composition data including total sugar, reducing sugar, nicotine, total nitrogen, potassium, and chlorine. However, when chemical composition combined with algorithmic models is used to characterize tobacco leaf positions, the acquisition of chemical components relies on traditional instrumental analytical methods, such as gas chromatography–mass spectrometry (GC–MS) and continuous flow analysis (CFA). As a result, the acquisition of chemical composition data is time-consuming and labor-intensive, involving only a limited number of compounds, and related research remains relatively scarce. SHapley Additive exPlanations (SHAP), derived from the game theory concept introduced by Lundberg and Lee, quantified the contribution of each feature to the model prediction (Anjum et al., 2022; Lu et al., 2023). Lu et al. (2023) established a stadium fire risk assessment model combined with the random forest model of SHAP strategy, and analyzed the impact of various risk characteristics on different risk assessment models. Long et al. (2024) used the SHAP model interpretation method to analyze the factors affecting the drug stability model. Santos et al. (2024) used SHAP model interpretation method to conduct feature screening in bearing fault diagnosis. Cui et al. (2024) built a landslide susceptibility evaluation model and revealed the influence of various influencing factors on the landslide susceptibility evaluation model through SHAP algorithm, thus enhancing the credibility and explainability of the model. Li S. et al. (2025) employed the SHAP model to quantify the contribution of individual aroma compounds to the machine learning prediction results, and identified key characteristic compounds that influence the sensory quality grade of sauce-flavor baijiu. These studies provide a reference for explaining the effect of chemical composition on tobacco leaf position discrimination model by SHAP algorithm. However, there is currently no report on using the SHAP method to analyze the chemical features in the tobacco leaf position discrimination model. Therefore, this study established a robust and accurate model for tobacco leaf position discrimination based on 70 chemical components. Significance analysis was conducted on chemical components across different leaf positions. The SHAP algorithm was employed to interpret the model and to analyze the influence of chemical components on position discrimination. This work provides valuable insights and references for accurately identifying tobacco leaf positions and analyzing their chemical characteristics.

## 2 Materials and methods

### 2.1 Materials

In China, tobacco leaves are classified based on stalk position, sub-grade, and color. The leaves are generally divided into three main stalk positions: upper (B), middle (C), and lower (X). Each position is further subdivided into three to four sub-grades, represented by the numbers 1, 2, 3, and 4. Common colors include orange-yellow (F), lemon-yellow (L), slightly greenish (V), and variegated (K).The sample set consisted of tobacco leaves from different positions (upper, middle, and lower) collected from 17 provinces in China, including Anhui, Fujian, Gansu, Guangxi, Guizhou, Henan, Heilongjiang, Hubei, Hunan, Jilin, Jiangxi, Liaoning, Shandong, Shaanxi, Sichuan, Yunnan, and Chongqing, with a total of 546 samples. Detailed sample information is shown in **Table 1**.

**Table 1:** Information on tobacco leaf samples.

Tobacco leaf position	Grade	Sample number	
Upper	B1F	25	206
	B2F	91	
	B3F	69	
	B4F	4	
	B1K	3	
	B2K	4	
	B3K	3	
	B2L	3	
	B3L	3	
	B2V	1	
Middle	C1F	18	270
	C2F	78	
	C3F	93	
	C4F	40	
	C1L	1	
	C2L	8	
	C3L	27	
	C4L	3	
	C3V	2	
Lower	X2F	54	70
	X3F	12	
	X4F	1	
	X2L	3	
Total			546

### 2.2 Acquisition and derivation of chemical components

All tobacco samples were dried in a drying room at 40°C for 1−3 days, ground to a certain granularity using a whirlwind grinding mill, and sieved through a 60-mesh sieve. The moisture content of the samples ranged between 6% and 8% and was analyzed by the oven-drying method. NIR spectra were recorded for all tobacco samples using the Antaris™ II Fourier Transform Near-Infrared (FT-NIR) spectrometer, equipped with an integrating sphere diffuse reflectance sampling system (Thermo Fisher Scientific, USA).Measurements were performed in triplicate, and each measurement comprised 64 co-added scans recorded at a resolution of 8 cm^−1^ in the wavenumber range of 4000−10000 cm^−1^.Multiplicative scatter correction (MSC) was performed prior to modeling to eliminate the uneven distribution of sample particles and reduce the effect of particle size on the spectra. The constant difference in the spectra was eliminated by taking the first derivative−because the calculation of the derivative tended to increase the noise−and performing Savitzky−Golay convolution smoothing prior to derivative preprocessing (Liang et al., 2022). In previous studies, our research team has proposed the near-infrared–chemical composition prediction model (Guo et al., 2023; Liang et al., 2022; Li B. et al., 2025). Based on the near-infrared–chemical composition prediction model, a total of 70 chemical components were identified, including routine chemical components of tobacco leaves, cations and anions, polyphenols, polyacids and higher fatty acids, amino acids and Amadori compounds, among others. The minimum and maximum values of the 70 chemical components in tobacco leaves can be found in the **Supplementary File**. These components encompass both major and trace substances in tobacco leaves and represent a crucial material foundation for tobacco leaf style and quality (**Table 2**).

**Table 2:** 70 chemical components in tobacco leaves.

No.	Type	Compound Name	Amount
1	Routine chemical components	Total alkaloids, Reducing sugar, Total sugar, Total nitrogen, Starch	5
2	Cations and anions	Potassium, Chlorine, Sulfate, Phosphate, Magnesium, Calcium	6
3	Polyphenols	Neo-chlorogenic acid, Chlorogenic acid, Cryptochlorogenic acid, Scopoletin, Rutin	5
4	Polyacids and higher fatty acids	Oxalic acid, Malonic acid, Succinic acid, Malic acid, Citric acid, Vanillic acid, Myristic acid, Palmitic acid, Linoleic acid, Oleic acid + Linolenic acid, Stearic acid, Arachidic acid	12
5	Amino acids	Aspartic acid, Threonine, Serine, Asparagine, Glutamic acid, Glutamine, Glycine, Alanine, Valine, Cystine, Methionine, Isoleucine, Leucine, Tyrosine, Phenylalanine, 4-Aminobutyric acid(GABA), Lysine, Histidine, Tryptophan, Arginine, Proline	21
6	Amadori compounds	N-(1-Deoxy-d-glucose-1-yl) Ammonia (Glu-An), N-(1-deoxy-D-fructos-1-yl) aminobutyric(Fru-Amb), N-(1-deoxy-D-fructos-1-yl) Histidine(Fru-His), N-(1-deoxy-D-fructos-1-yl) Proline(Fru-Pro), N-(1-deoxy-D-fructos-1-yl) Valine(Fru-Val), N-(1-deoxy-D-fructos-1-yl) Threonine(Fru-Thr), N-(1-deoxy-D-fructos-1-yl) Glycine(Fru-Gly), N-(1-deoxy-D-fructos-1-yl) Alanine(Fru-Ala), N-(1-deoxy-D-fructos-1-yl) Asparagine(Fru-Asn), N-(1-deoxy-D-fructos-1-yl) Asparticacid(Fru-Asp), N-(1-deoxy-D-fructos-1-yl) Glutarnine(Fru-Gln), N-(1-deoxy-D-fructos-1-yl) Glutamicacid(Fru-Glu), N-(1-deoxy-D-fructos-1-yl) Isoleucine(Fru-Ile), N-(1-deoxy-D-fructos-1-yl) Leucine(Fru-Leu), N-(1-deoxy-D-fructos-1-yl) Tyrosine(Fru-Tyr), N-(1-deoxy-D-fructos-1-yl) Phenylalanine(Fru-Phe), N-(1-deoxy-D-fructos-1-yl) Tryptophan(Fru-Trp)	17
7	Others	pH, Dichloromethane extract, Solanesol, Neo-phytene	4
Total			70

Tobacco, as an important economic crop, has its industrial usability partly influenced by the balanced proportions of chemical components such as total alkaloids, total nitrogen, potassium, and chlorine. As a result, derived indexes such as the sugar-alkaloid ratio, nitrogen-alkaloid ratio, and potassium-chlorine ratio have been developed. These derived indexes can, in some cases, more directly reflect the quality of tobacco leaves (Lü et al., 2020).The derivatives of cations and anions, polyphenols, polyacids and higher fatty acids, amino acids, Amadori compounds were obtained by addition. Derivatization indexes such as sugar-alkaloid ratio, nitrogen-alkaloid ratio, schmuck value (Sugar-to-protein ratio), and potassium-chlorine ratio were obtained by the ratio. The specific information is shown in **Table 3**.

**Table 3:** 9 derivatization indexes.

No.	Derived indexes
1	Sugar-alkaloid ratio
2	Nitrogen-alkaloid ratio
3	Schmuck value
4	Potassium-chlorine ratio
5	Cations and anions
6	Polyphenols
7	Polyacids and higher fatty acids
8	Amino acids
9	Amadori compounds

### 2.3 Model construction and interpretation

**Figure 1:**
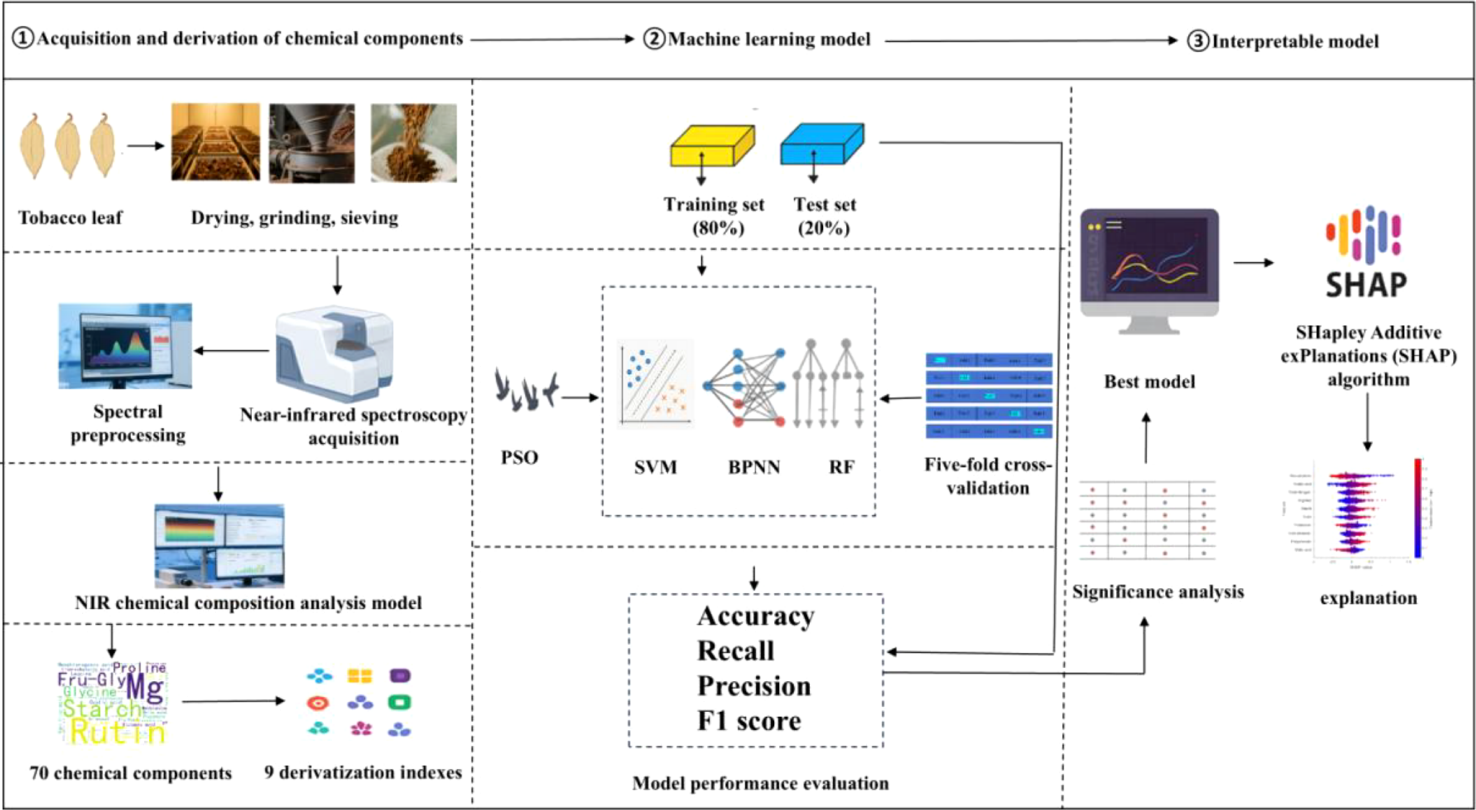
Flowchart of model construction and interpretation.

As shown in **Figure 1**, model construction and interpretation are carried out.

#### 2.3.1 Five-fold cross-validation and external validation

Stratified sampling was used to divide tobacco samples into the training set (80%) and the independent test set (20%) without overlapping data. Five-fold cross-validation was applied within the training set to determine optimal parameters, while the independent test set was excluded from the cross-validation process. After optimizing each model and identifying the best parameters, the entire training set (80%) was used again to retrain the model, and the independent test set (20%) was used for final performance evaluation.

#### 2.3.2 Model construction

**Figure 2:**
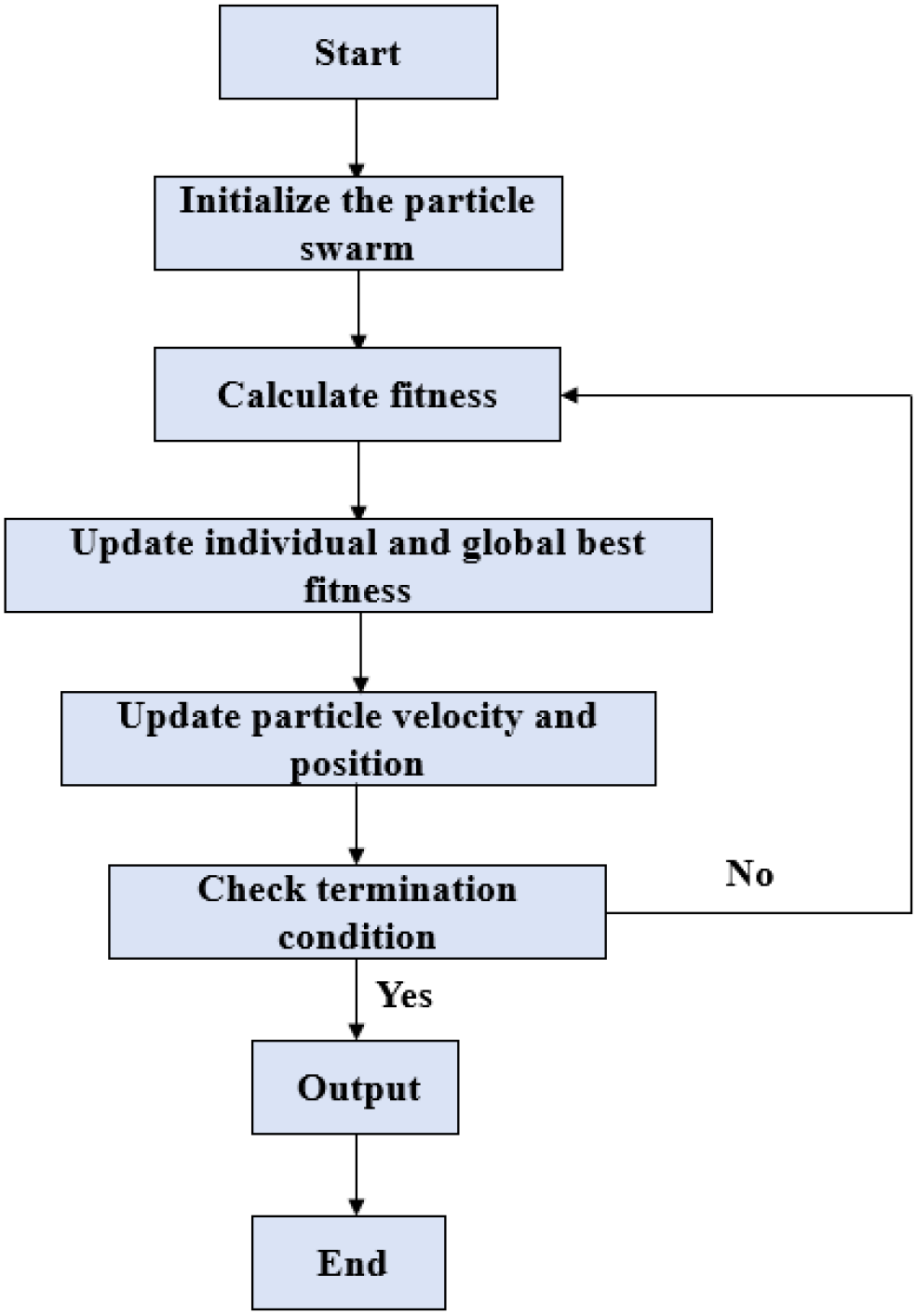
Particle swarm optimization (PSO) algorithm flowchart.

Support vector machine (SVM), back propagation neural network (BPNN) and random forest (RF) were used to construct the tobacco leaf position discrimination model. In order to improve the classification performance of each model, particle swarm optimization (PSO) was used to find the optimal parameter combination for the performance of each model. Particle swarm optimization (PSO) is a swarm based random optimization algorithm, inspired by the intelligent collective behavior of some animals (such as flocks of birds or fish). Due to its advantages such as fast convergence speed, strong global search ability and strong adaptability, PSO is widely used in optimization tasks of machine learning (Wang et al., 2018b; Xu et al., 2024; Li et al., 2024; Zhu et al., 2024). The flow of the Particle Swarm Optimization (PSO) algorithm is shown in **Figure 2**.

The inertia weight in the PSO algorithm was set to 0.9 (linearly decayed to 0.4), the acceleration constants *c*_1_ and *c*_2_ were both 1.5, and the random factors *r*_1_ and *r*_2_ were randomly generated in the range [0, 1]. The average accuracy of five-fold cross-validation was used as the fitness function and applied to the parameter optimization of SVM, BPNN and RF models. In the particle swarm optimization algorithm of SVM model, the influence of different kernel functions and their parameter settings on classification performance is emphasized. The parameter settings of each kernel function are shown in **Table 4**, and the parameter range of particle swarm optimization is shown in **Table 5**. In the particle swarm optimization algorithm of BPNN model, key parameters such as the number of hidden layers, the number of neurons in each layer and the learning rate are adjusted. The tuning range of particle swarm optimization is shown in **Table 5**. In the particle swarm optimization algorithm of the RF model, key parameters such as the number of trees and the minimum number of samples required for each leaf node are adjusted. The range of particle swarm optimization parameters is shown in **Table 5**.

**Table 4:** Parameter settings for the SVM kernel function.

Kernel function	Parameter setting
linear kernel	*y*= $x_{1}\cdot x_{2}^{'}$
polynomial kernel	$y={\left(c_{1}\cdot x_{1}\cdot x_{2}^{"}+c_{2}\right)}^{c_{3}}$
Gaussian kernel	$y=\text{exp}(-\frac{\left(x_{1}-x_{2}\right)\left(x_{1}-x_{2}\right)"}{\sigma^{2}})$
sigmoid kernel	$y=\text{tanh}\left(a\cdot x_{1}\cdot x_{2}^{'}+c\right)$
hybrid kernel	y _0_= $x_{1}\cdot x_{2}^{'}$
	*y*_1_= ${\left(c_{1}\cdot x_{1}\cdot x_{2}^{'}+c_{2}\right)}^{c_{3}}$
	*y*_2_= $\text{ exp}(-\frac{\left(x_{1}-x_{2}\right)\left(x_{1}-x_{2}\right)"}{\sigma^{2}})$
	*y*=*my*_0_+*ny*_1_+*qy*_2_

*c*_1_: control the weight of the polynomial kernel; *c*_2_: control the strength of the offset item and affect the mapping of the data; *c*_3_: the order of the polynomial, which controls the dimension of the map; *σ²*: determines the width of the Gaussian function and affects the range of the kernel function; *a*: the parameters of the kernel function control the influence of the inner product; *c*: constant term, used to adjust the offset of the function; *m*, *n*, *q*: control the weighting coefficient of each kernel function and determine the contribution of each kernel function.

**Table 5:** Particle swarm optimization tuning range.

Model	Tuning range
SVM- linear kernel	/
SVM- polynomial kernel	*c*_1_: [1/80, 1/40]; *c*_2_: [0.1, 3]; *c*_3_: [1, 4]
SVM- Gaussian kernel	*σ²*: [10, 50]
SVM-sigmoid kernel	*a*: [1/100, 1]; *c*: [-5, 5]
SVM- hybrid kernel	*c*_1_: [1/80, 1/40]; *c*_2_: [0.1, 3]; *c*_3_: [1, 4]
	*σ²*: [10, 50]
	*m*, *n*, *q*: [0, 1]; *m* + *n* + *q* = 1
BPNN	the number of hidden layers: [1, 3]
	the number of neurons in each layer: [2, 60]
	the learning rate: [0.001, 0.1]
RF	the number of trees: [50, 200]
	the minimum number of samples required for each leaf node: [2, 10]

#### 2.3.3 Model performance evaluation

The accuracy (Acc), recall (R), precision (P), F1 score (F1), macro-average recall (macro-R), macro-average precision (macro-P), macro-average F1 score (macro-F1) and other indicators were selected to evaluate the model performance. The calculation method is shown in Equations 1-7.


(1) $$Acc=\frac{\sum_{i=1}^{n}TP_{i}}{\sum_{i=1}^{n}\left(TP_{i}+FP_{i}\right)}$$

(2) $$R=\frac{TP}{TP+FN}$$

(3) $$P=\frac{TP}{TP+FP}$$

(4) $$F1=\frac{2\times P\times R}{P+R}$$

(5) $$macro-R=\frac{1}{n}\sum_{i=1}^{n}\frac{TP_{i}}{TP_{i}+FN_{i}}$$

(6) $$macro-P=\frac{1}{n}\sum_{i=1}^{n}\frac{TP_{i}}{TP_{i}+FP_{i}}\;$$

(7) $$macro-F1=\frac{2\times macro-R\times macro-P}{macro-R+macro-P}\;$$

In the formula: *TP* represents the number of samples predicted to be positive in fact; *FN* represents the number of samples that are actually positive and predicted to be negative. *FP* represents the number of samples that are actually negative and predicted to be positive.

#### 2.3.4 Significance analysis of chemical indexes in different tobacco positions

Independent sample t-test was used to analyze whether there were significant differences between the mean values of each chemical index between the upper tobacco leaves and the leaves of other positions (middle and lower), the middle tobacco leaves and the leaves of other positions (upper and lower), and the lower tobacco leaves and the leaves of other positions (upper and middle). To reduce the risk of false positives and improve the rigor of the statistical test results, the significance level was set at 0.0167 (0.05/3).If the p-value is lower than the significance level, it is considered that there are significant differences between the mean values of the two groups. Otherwise, the difference is not considered significant.

#### 2.3.5 Model interpretation

SHapley Additive exPlanations (SHAP) algorithm was used to explain the best model. The core idea of SHAP algorithm is derived from Shapley value in game theory. The Shapley value was originally used to assign the value contributed by each player in a cooperative game. In machine learning models, SHAP values can be used to explain the contribution of each feature to the model’s predictions. The Shapley value can fairly assign the contribution of each feature to the model prediction results. The basic principle of its calculation is to consider all possible feature subsets for each feature and calculate the change in model performance when the feature is added, that is, the marginal contribution of the feature to the model prediction results. The calculation method of Shapley value is shown in Equation 8.


(8) $$\Phi i\;=\sum_{S\subseteq N\setminus \left\{i\right\}}\frac{\left|S\right|!\left(\left|F\right|-\left|S\right|-1\right)!}{\left|F\right|!}\left[f\left(S^{\cup }\left\{i\right\}\right)-\;f\left(S\right)\right]$$

In the formula: *Φ_i_* is the Shapley value of feature *i*; *F* is the global feature set; *S* is a subset without feature *i*; *f(S)* is the predicted value of the model when using subset *S*.

SHAP algorithm can quantify the contribution of each chemical index to tobacco leaf position discrimination model, and is suitable for various types of machine learning models, and has wide applicability in different tobacco leaf position discrimination model research. At the same time, SHAP algorithm has many advantages, such as clarifying the importance of features to the model and their impact on the entire prediction model, and understanding the contribution of each feature to the model output (Rodriguez-Perez and Bajorath, 2020). In this study, SHAP feature importance analysis, SHAP summary plots and SHAP dependence plots were used to clarify the internal working principle of the model, analyze the contribution of features to the model prediction, and thus improve the transparency and interpretability of the discrimination model.

#### 2.3.6 Software

MATLAB (version R2022a) is used to implement the data processing and analysis process.

## 3 Results

### 3.1 Model construction and evaluation

Based on tobacco leaf sample set, particle swarm optimization algorithm was used to find the optimal parameter combination of SVM, BPNN and RF machine learning models. **Table 6** presents the optimal parameters obtained for each model through particle swarm optimization (PSO), using the average accuracy of five-fold cross-validation as the fitness function. **Table 7** presents the validation accuracy of each fold and the average accuracy of five-fold cross-validation for each model under the optimal parameters. For SVM model, hybrid kernel function was the most prominent, and the average accuracy of five-fold cross-validation was the highest. The possible reason is that the hybrid kernel function can combine the advantages of multiple kernel functions, and give full play to the advantages of each kernel function through the weighted combination of each kernel function to improve the classification ability of the model. Therefore, the hybrid kernel was determined as the final kernel function of SVM. For the BPNN model, when the hidden layer was 1, the number of neurons in each hidden layer was 30, and the learning rate was 0.01, the average accuracy of five-fold cross-validation was the highest. The possible reason is that for a dataset with limited samples, multiple hidden layers and a large number of neurons may cause the model to be too complicated, thus leading to overfitting. However, the setting of 1 hidden layer, 30 neurons and the learning rate of 0.01 may just balance the complexity and generalization ability of the model, avoiding overfitting and underfitting. For the RF model, when the number of trees was 100 and the minimum sample number of leaf nodes was 5, the average accuracy of the five-fold cross-validation was the highest. The possible reason is that this setting can balance the complexity and computational efficiency of the model, avoid over-fitting and improve the stability and generalization ability of the model.

**Table 6:** The optimal parameters obtained for each model through particle swarm optimization (PSO).

Model	Optimal parameter
SVM- linear kernel	/
SVM- polynomial kernel	*c*_1_ = 1/60,*c*_2_ = 1,*c*_3_ = 3
SVM- Gaussian kernel	*σ²*=18
SVM-sigmoid kernel	*a*=1/60, *c*= -1.5
SVM- hybrid kernel	*c*_1_ = 1/66,*c*_2_ = 1.9,*c*_3_ = 3
	*σ^2^ =* 30
	*m*=0,*n*=0.5,*q*=0.5
BPNN	the number of hidden layers =1, the number of neurons in each layer =30, the learning rate =0.01
RF	the number of trees =100, the minimum number of samples required for each leaf node =5

**Table 7:** The validation accuracy of each fold and the average accuracy of five-fold cross-validation for each model under the optimal parameters.

Model	Fold 1/%	Fold 2/%	Fold 3/%	Fold 4/%	Fold 5/%	Average/%
SVM- linear kernel	91.95	87.50	92.05	85.06	80.46	87.40
SVM- polynomial kernel	87.36	90.91	88.64	87.36	83.91	87.63
SVM- Gaussian kernel	75.86	82.95	89.77	79.31	82.76	82.13
SVM-sigmoid kernel	77.01	88.64	86.36	77.01	80.46	81.90
SVM- hybrid kernel	87.36	90.91	89.77	89.66	87.36	89.01
BPNN	87.36	89.77	86.36	83.91	80.46	85.57
RF	85.06	85.23	87.50	81.61	81.61	84.20

After optimizing each model and determining the optimal parameters, the models were trained using the training set, while the test set was used for performance evaluation. **Table 8** presents the discrimination accuracy of the training and test sets for each model. The SVM-hybrid kernel achieved the highest accuracy on both the training and test sets, reaching 98.17% and 96.33%, respectively.

**Table 8:** The discrimination accuracy of the training and test sets for each model.

Model	Training set accuracy/%	Test set accuracy/%
SVM-hybrid kernel	98.17	96.33
BPNN	93.59	88.07
RF	97.25	92.66

**Tables 9**, **10** present the recall, precision, and *F*1 score of each model for each tobacco leaf position, as well as the macro-average recall, macro-average precision, and macro-average F1 score. The SVM-hybrid kernel demonstrated the best performance, with the macro-average recall, macro-average precision, and macro-average *F*1 score of 0.9368, 0.9522, and 0.9444, respectively. Its results were the most stable and accurate, indicating strong robustness.

**Table 9:** The recall, precision, and *F*1 score of each model for each tobacco leaf position.

Model	Recall			precision			*F*1 score		
	Upper	Middle	Lower	Upper	Middle	Lower	Upper	Middle	Lower
SVM-hybrid kernel	1.0000	0.9643	0.8462	0.9756	0.9643	0.9167	0.9877	0.9643	0.8800
BPNN	0.9250	0.8929	0.6923	1.0000	0.8772	0.6000	0.9610	0.8850	0.6429
RF	0.9750	0.9464	0.6923	0.9512	0.9138	0.9000	0.9630	0.9298	0.7826

**Table 10:** The macro-average recall, macro-average precision, and macro-average *F*1 score of each model.

Model	Macro-average recall	Macro-average precision	Macro-average *F*1 score
SVM-hybrid kernel	0.9368	0.9522	0.9444
BPNN	0.8367	0.8257	0.8312
RF	0.8712	0.9217	0.8957

**Table 11** shows the confusion matrix results of the SVM-hybrid kernel model in the test set. Except that one middle leaf was misjudged as the upper leaf, one middle leaf was misjudged as the lower leaf, and two lower leaves were misjudged as the middle leaf, all the other samples were correctly judged. Analysis of the error samples showed that the sample wrongly identified as the upper tobacco leaf was grade C_1_F tobacco leaf, which was incorrectly identified by the model because of its proximity to the upper tobacco leaf. The sample wrongly identified as the lower tobacco leaf was grade C_4_F tobacco leaf, which was incorrectly identified by the model because of its proximity to the lower tobacco leaf.

**Table 11:** The confusion matrix results of the SVM-hybrid kernel model in the test set.

Test set	Tobacco leaf position		
	Upper	Middle	Lower
Upper	40	0	0
Middle	1	54	1
Lower	0	2	11

### 3.2 Significance analysis of chemical indexes in different tobacco positions

Independent sample t-test was used to analyze whether there were significant differences between the mean values of each chemical index between the upper tobacco leaves and the leaves of other positions (middle and lower), the middle tobacco leaves and the leaves of other positions (upper and lower), and the lower tobacco leaves and the leaves of other positions (upper and middle). As presented in **Table 12**, the results indicate that there were significant differences in 62 chemical indices between the upper tobacco leaves and those from other positions (middle and lower), while 17chemical indices showed no significant differences. Additionally, a comparison of the middle tobacco leaves with those from other positions (upper and lower) revealed significant differences in 62 chemical indices, whereas 17 chemical indices exhibited no significant differences. Furthermore, when comparing the lower tobacco leaves to those from other positions (upper and middle), it was found that there were significant differences in 58 chemical indices and no significant differences in 21 chemical indices.

**Table 12:** Results of independent sample t-test for each chemical index in different tobacco positions.

Chemical index	Upper leaves vs. other leaves			Middle leaves vs. other leaves			Lower leaves vs. other leaves		
	t-value	degree of freedom	p-value	t-value	degree of freedom	p-value	t-value	degree of freedom	p-value
Total alkaloids	23.63	544.00	0.00	-10.07	434.71	0.00	-18.17	157.92	0.00
Reducing sugar	-8.81	544.00	0.00	11.21	520.52	0.00	-3.16	544.00	0.00
Total sugar	-10.75	407.45	0.00	12.50	519.12	0.00	-2.08	544.00	0.04
Total nitrogen	22.79	544.00	0.00	-13.10	488.11	0.00	-9.58	126.44	0.00
Potassium	-10.58	519.67	0.00	3.11	518.79	0.00	7.14	79.38	0.00
Chlorine	0.45	544.00	0.65	-1.52	544.00	0.13	1.62	544.00	0.11
pH	-7.41	383.36	0.00	2.92	516.11	0.00	6.51	544.00	0.00
Starch	1.81	544.00	0.07	4.00	532.75	0.00	-9.13	544.00	0.00
Dichloromethane extract	13.31	299.36	0.00	-9.64	451.40	0.00	-7.30	134.26	0.00
Solanesol	14.10	334.66	0.00	-9.79	480.02	0.00	-6.69	114.85	0.00
Sulfate	1.53	544.00	0.13	-0.93	544.00	0.35	-0.83	544.00	0.41
Phosphate	5.85	544.00	0.00	-4.32	544.00	0.00	-1.63	83.14	0.11
Magnesium	1.45	544.00	0.15	-4.84	484.19	0.00	3.82	78.58	0.00
Calcium	-1.89	503.63	0.06	-2.57	544.00	0.01	5.69	82.71	0.00
Neo-chlorogenic acid	-12.76	544.00	0.00	5.15	494.76	0.00	9.43	544.00	0.00
Chlorogenic acid	-1.28	370.03	0.20	2.50	526.06	0.01	-1.77	544.00	0.08
Cryptochlorogenic acid	-13.85	544.00	0.00	5.37	501.82	0.00	12.01	101.77	0.00
Scopoletin	13.51	304.01	0.00	-12.85	455.58	0.00	-2.05	116.54	0.04
Rutin	7.42	544.00	0.00	-2.18	530.50	0.03	-8.54	101.89	0.00
Oxalic acid	6.72	544.00	0.00	-8.43	540.54	0.00	2.50	544.00	0.01
Malonic acid	15.47	353.44	0.00	-11.98	492.46	0.00	-4.44	106.83	0.00
Succinic acid	-10.82	541.87	0.00	0.36	472.66	0.72	10.58	77.10	0.00
Malic acid	-5.48	501.73	0.00	-1.57	528.31	0.12	9.05	82.52	0.00
Citric acid	3.02	544.00	0.00	-7.90	451.44	0.00	4.51	74.61	0.00
Vanillic acid	19.50	330.77	0.00	-11.35	440.03	0.00	-12.26	150.93	0.00
Myristic acid	20.01	365.08	0.00	-11.40	456.02	0.00	-10.39	118.82	0.00
Palmitic acid	-9.03	544.00	0.00	7.99	532.00	0.00	0.76	81.04	0.45
Linoleic acid	13.68	321.58	0.00	-7.22	439.56	0.00	-11.72	124.23	0.00
Oleic acid + Linolenic acid	-15.00	507.18	0.00	9.00	544.00	0.00	5.12	544.00	0.00
Stearic acid	-14.10	544.00	0.00	9.37	518.69	0.00	3.98	83.16	0.00
Arachidic acid	-6.98	528.73	0.00	-1.31	544.00	0.19	11.03	84.36	0.00
Aspartic acid	-0.04	535.73	0.97	-6.64	497.16	0.00	6.68	74.67	0.00
Threonine	3.19	544.00	0.00	-7.76	492.12	0.00	4.53	76.11	0.00
Serine	-0.32	544.00	0.75	-1.96	533.23	0.05	3.41	544.00	0.00
Asparagine	7.83	544.00	0.00	-11.19	447.13	0.00	3.05	76.86	0.00
Glutamic acid	4.69	544.00	0.00	-10.28	478.54	0.00	5.43	76.73	0.00
Glutamine	7.59	544.00	0.00	-9.57	501.76	0.00	2.36	82.87	0.02
Glycine	11.77	544.00	0.00	-12.89	500.60	0.00	1.60	544.00	0.11
Alanine	8.60	544.00	0.00	-9.27	544.00	0.00	1.27	94.81	0.21
Valine	-5.76	544.00	0.00	2.73	528.61	0.01	3.41	81.53	0.00
Cystine	11.74	544.00	0.00	-7.88	544.00	0.00	-4.09	544.00	0.00
Methionine	3.11	544.00	0.00	-6.16	507.12	0.00	3.80	82.36	0.00
Isoleucine	1.85	544.00	0.07	-4.52	523.66	0.00	3.09	79.33	0.00
Leucine	0.70	544.00	0.48	-1.23	544.00	0.22	0.83	544.00	0.41
Tyrosine	2.07	544.00	0.04	-6.58	508.49	0.00	4.53	75.85	0.00
Phenylalanine	4.25	502.70	0.00	-8.88	508.62	0.00	4.76	76.35	0.00
4-Aminobutyric acid (GABA)	5.87	544.00	0.00	-9.91	506.61	0.00	4.33	79.96	0.00
Lysine	11.33	544.00	0.00	-12.02	490.21	0.00	0.97	82.05	0.34
Histidine	7.58	544.00	0.00	-10.43	501.54	0.00	3.02	80.25	0.00
Tryptophan	5.95	544.00	0.00	-8.36	544.00	0.00	2.97	83.10	0.00
Arginine	15.46	544.00	0.00	-13.63	495.45	0.00	-1.14	544.00	0.26
Proline	10.86	544.00	0.00	-5.92	544.00	0.00	-5.88	544.00	0.00
Glu-An	13.21	396.08	0.00	-12.18	522.89	0.00	-0.90	544.00	0.37
Fru-Amb	-9.07	544.00	0.00	5.78	531.81	0.00	3.94	544.00	0.00
Fru-His	4.50	544.00	0.00	-2.53	544.00	0.01	-2.66	544.00	0.01
Fru-Pro	1.25	544.00	0.21	4.65	525.34	0.00	-9.27	544.00	0.00
Fru-Val	-10.08	507.60	0.00	4.91	544.00	0.00	5.77	544.00	0.00
Fru-Thr	-11.47	396.94	0.00	5.21	507.04	0.00	9.33	101.01	0.00
Fru-Gly	6.17	544.00	0.00	-1.68	531.52	0.09	-6.38	544.00	0.00
Fru-Ala	-5.02	544.00	0.00	8.53	533.26	0.00	-4.07	81.27	0.00
Fru-Asn	5.65	544.00	0.00	-5.44	544.00	0.00	-0.04	544.00	0.97
Fru-Asp	-11.93	507.98	0.00	5.74	541.12	0.00	6.63	544.00	0.00
Fru-Gln	-0.48	402.28	0.63	-0.83	544.00	0.41	2.26	101.27	0.03
Fru-Glu	-6.51	544.00	0.00	1.83	526.96	0.07	6.63	544.00	0.00
Fru-Ile	-5.12	527.35	0.00	1.85	544.00	0.07	4.04	544.00	0.00
Fru-Leu	-6.04	544.00	0.00	3.35	544.00	0.00	3.56	544.00	0.00
Fru-Tyr	-7.14	544.00	0.00	5.71	543.27	0.00	1.62	544.00	0.11
Fru-Phe	-6.39	383.86	0.00	3.80	528.20	0.00	3.69	544.00	0.00
Fru-Trp	0.22	544.00	0.83	2.06	544.00	0.04	-3.43	544.00	0.00
Neo-phytene	-4.17	544.00	0.00	-0.27	540.06	0.78	6.61	544.00	0.00
Sugar-alkaloid ratio	-19.47	543.79	0.00	8.31	524.47	0.00	9.07	544.00	0.00
Nitrogen-alkaloid ratio	-14.44	494.55	0.00	1.58	425.22	0.12	9.31	73.06	0.00
Schmuck value	-5.98	537.04	0.00	3.69	515.33	0.00	1.49	79.44	0.14
Potassium-chlorine ratio	-15.33	544.00	0.00	14.59	544.00	0.00	0.08	544.00	0.94
Cations and anions	0.88	544.00	0.38	-1.82	544.00	0.07	1.44	544.00	0.15
Polyphenols	1.08	357.51	0.28	0.94	526.01	0.35	-3.09	544.00	0.00
Polyacids and higher fatty acids	-3.29	499.44	0.00	-3.19	530.67	0.00	8.26	81.45	0.00
Amino acids	11.05	544.00	0.00	-9.25	536.79	0.00	-1.64	544.00	0.10
Amadori compounds	-0.74	544.00	0.46	4.21	517.27	0.00	-5.24	544.00	0.00

### 3.3 Interpretation of tobacco leaf position discrimination model by SVM- hybrid kernel based on SHAP algorithm

The mean difference of chemical indexes in different positions of tobacco leaves was analyzed by independent sample t-test, and the chemical indexes with significant differences in different positions were identified. There were significant differences in 62 chemical indexes between the upper and other positions of tobacco leaves (middle and lower), 62 chemical indexes between the middle and other positions of tobacco leaves (upper and lower), and 58 chemical indexes between the lower and other positions of tobacco leaves (upper and middle). These chemical indexes with significant differences may play an important role in the discrimination of tobacco leaf positions. However, independent sample t-test only provides the significance of mean differences, and cannot give the importance ranking and contribution degree of chemical indexes to model discrimination, nor can it quantify the specific contribution of each chemical index to the discrimination model. SHAP algorithm can quantify the importance of each feature in model discrimination, and assign a contribution value to each feature to intuitively show the specific impact of features in different categories.

Therefore, SHAP algorithm is used to interpret the SVM- hybrid kernel model. Since SVM is essentially a binary classification model that finds a separated hyperplane by maximizing the spacing between classes, for a multi-classification problem, SVM splits it into multiple binary classification problems, each class being compared to all the others. When applying SHAP to the SVM model, SHAP calculates the contribution of each feature to each category prediction, that is, the contribution of each feature to the separation boundary between the category and the other categories. Therefore, when the SHAP algorithm is used for model interpretation, it will be divided into the interpretation of the upper tobacco leaf to the other positions of tobacco leaves (middle and lower tobacco leaves), the interpretation of the middle tobacco leaf to the other positions of tobacco leaves (upper and lower tobacco leaves), and the interpretation of the lower tobacco leaf to the other positions of tobacco leaves (upper and middle tobacco leaves).

#### 3.3.1 Interpretation of the discrimination model of the upper tobacco leaves to the other positions of tobacco leaves (middle and lower)

**Figure 3:**
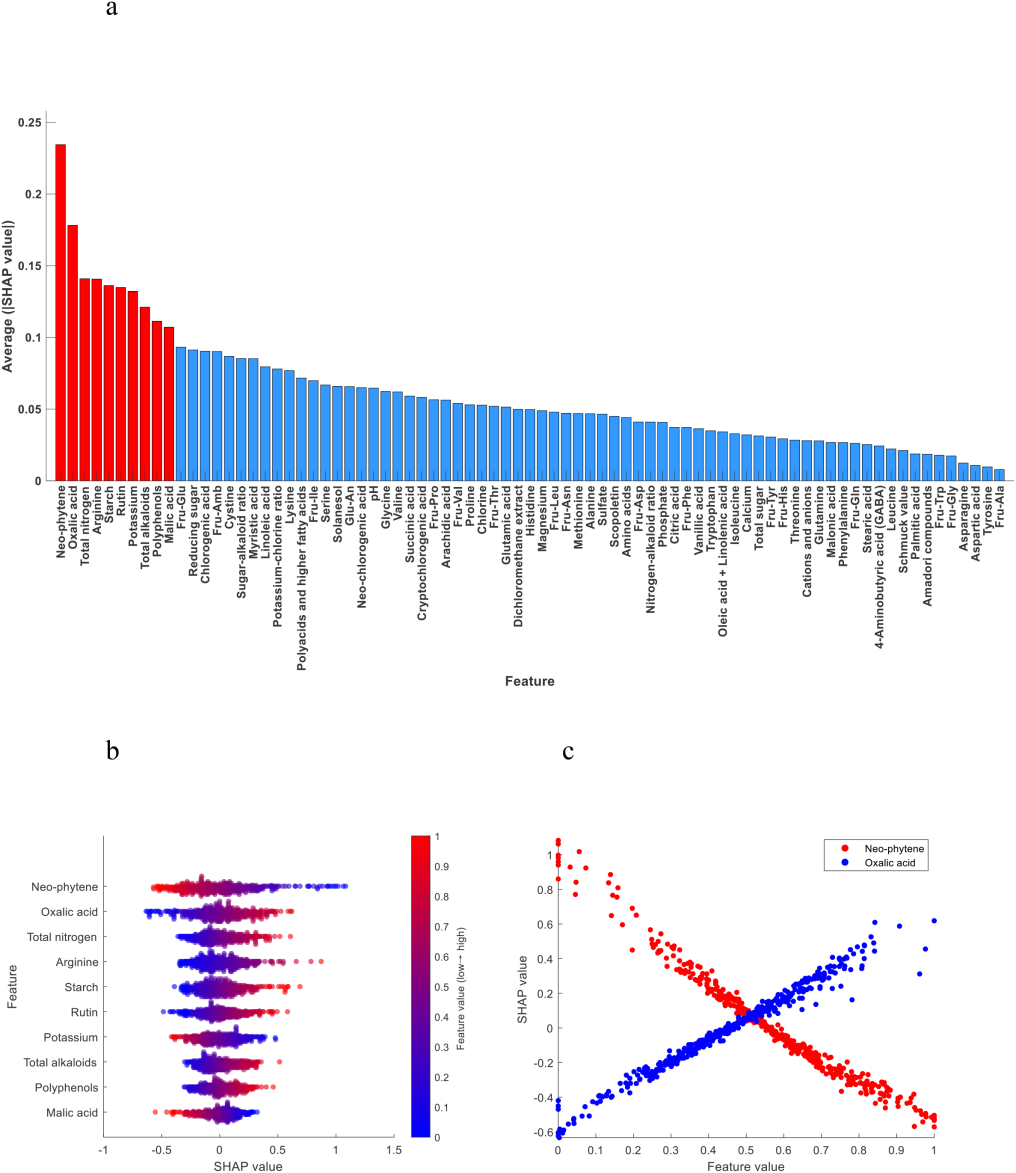
**(a)** Importance ranking of chemical indexes; **(b)** SHAP summary plot of the top 10 chemical indexes; **(c)** SHAP dependence plot of neo-phytene and oxalic acid.

The SHAP value is used to quantify the impact of each feature on the model output, and its value reflects the specific contribution of that feature to the model prediction. In model prediction, each feature has a different degree of contribution, and the SHAP value assigns the marginal contribution of each feature to the predicted outcome by using the Shapley value method in game theory. The absolute value of SHAP of each chemical index in the model is averaged, and then sorted in descending order to obtain the ranking of feature importance (Cui et al., 2024). As shown in **Figure 3a**, the top 10 most important chemical indexes in the discrimination model of the upper tobacco leaves to the other positions of tobacco leaves (middle and lower)were neo-phytene, oxalic acid, total nitrogen, arginine, starch, rutin, potassium, total alkaloids, polyphenols and malic acid.

The SHAP summary plot shows the influence of features (chemical indexes) on the model prediction, where each point represents a real sample, and the X-axis represents the SHAP value, representing the contribution degree and influence of features (chemical indexes) on the model prediction. Positive values represent positive impacts, that is, positive contributions, and negative values represent negative impacts, that is, negative contributions. The color gradient (blue to red) indicates the content level of the chemical index from low to high after normalization. The color of the points reflects the content level of the chemical index. The higher the content, the redder the color, and the lower the content, the bluer the color. The SHAP summary plot shows a gradual transition from left to right, from blue to red, indicating that with the increase of the content of this category of chemical index in tobacco leaves, the sample was more inclined to be awarded to this category, that is, the chemical index contributed positively to the model discrimination. On the contrary, the transition from red to blue indicates that with the increase of the content of this chemical index in tobacco leaves, the samples are more inclined to be awarded to other categories, indicating that this chemical index has a negative contribution to model discrimination.

**Figure 3b** shows the SHAP summary plot of the top 10 most important chemical indexes. We found that oxalic acid, total nitrogen, arginine, starch, rutin, total alkaloids and polyphenols contributed positively to the model discrimination, that is, with the increase of the content, the model tended to classify tobacco leaves as the upper leaves. Neo-phytene, potassium and malic acid contributed negatively to the model discrimination, that is, with the increase of content, the model tended to classify tobacco leaves as other positions.

In order to more clearly analyze the influence of chemical indexes on the model discrimination results, the SHAP dependence plot of each chemical index was obtained after the chemical indexes were normalized. Taking neo-phytene and oxalic acid as examples, as shown in **Figure 3c**, with the increase of neo-phytene content, the SHAP value gradually decreased, and the model tended to classify tobacco leaves as other positions. With the increase of oxalic acid content, the SHAP value also gradually increased, and the model was more tended to classify tobacco leaves as upper leaves.

#### 3.3.2 Interpretation of the discrimination model of the middle tobacco leaves to the other positions of tobacco leaves (upper and lower)

**Figure 4:**
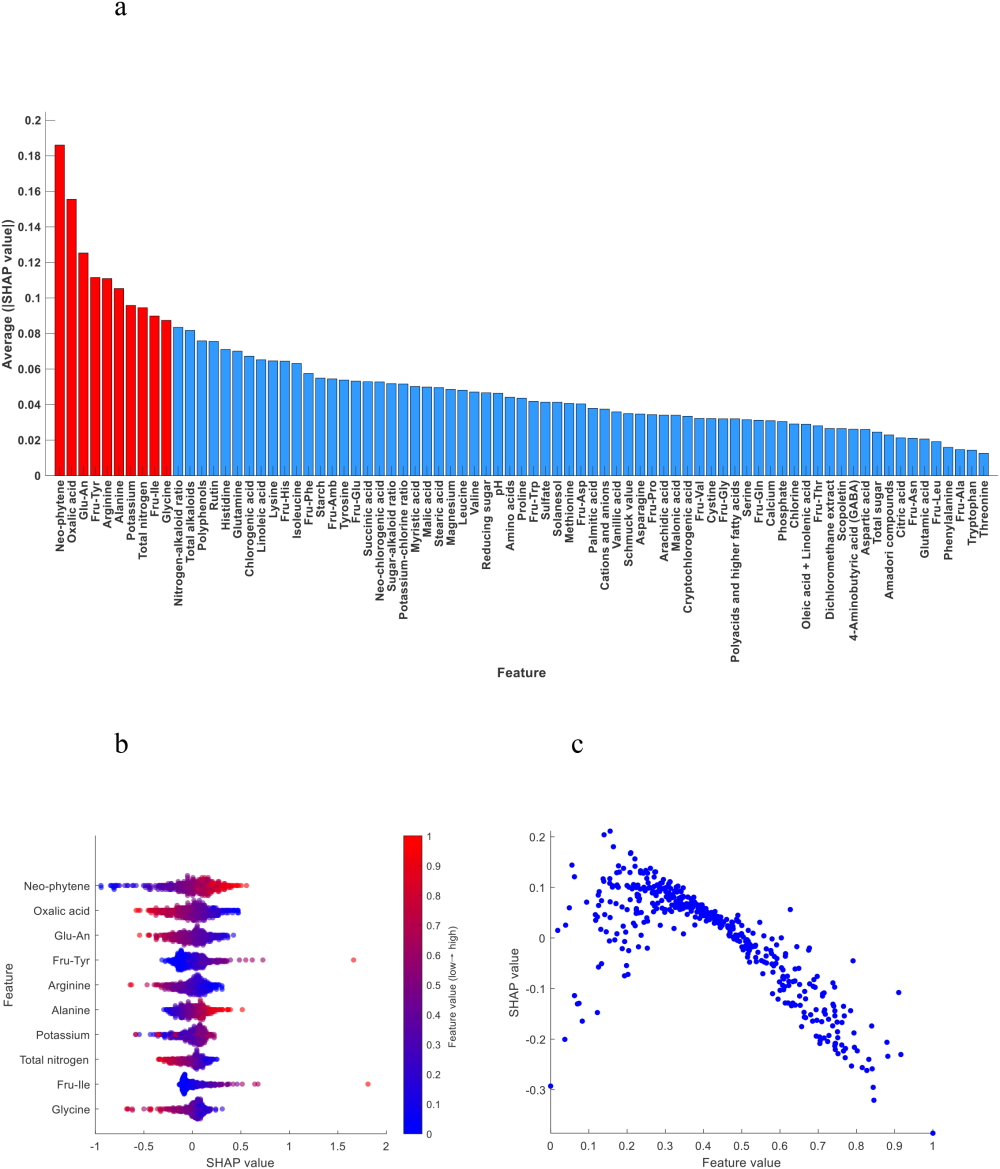
**(a)** Importance ranking of chemical indexes; **(b)** SHAP summary plot of the top 10 chemical indexes; **(c)** SHAP dependence plot of total alkaloids.

As shown in **Figure 4a**, the top 10 most important chemical indexes in the discrimination model of the middle tobacco leaves to the other positions of tobacco leaves (upper and lower) were neo-phytene, oxalic acid, Glu-An, Fru-Tyr, arginine, alanine, potassium, total nitrogen, Fru-Ile and glycine.

**Figure 4b** shows the SHAP summary plot of the top 10 most important chemical indexes. We found that neo-phytene, Fru-Tyr, alanine, potassium and Fru-Ile contributed positively to the model discrimination, that is, with the increase of the content, the model tended to classify tobacco leaves as the middle leaves. Oxalic acid, Glu-An, arginine, total nitrogen and glycine contributed negatively to the model discrimination, that is, with the increase of content, the model tended to classify tobacco leaves as other positions.

At the same time, it was found that some features in the SHAP dependence plot showed quadratic distribution, taking the total alkaloids as an example (**Figure 4c**). In the discrimination model of the middle tobacco leaves to the other positions of tobacco leaves (upper and lower), when the total alkaloids content was moderate, SHAP value was higher, that is, the model tended to classify tobacco leaves as middle leaves. With too high or too low total alkaloids content, the model tends to classify tobacco leaves as other positions. This is also consistent with previous studies (Wang et al., 2009; Zhang et al., 2022), that is, the total alkaloids content of middle tobacco leaves is moderate.

#### 3.3.3 Interpretation of the discrimination model of the lower tobacco leaves to the other positions of tobacco leaves (upper and middle)

**Figure 5:**
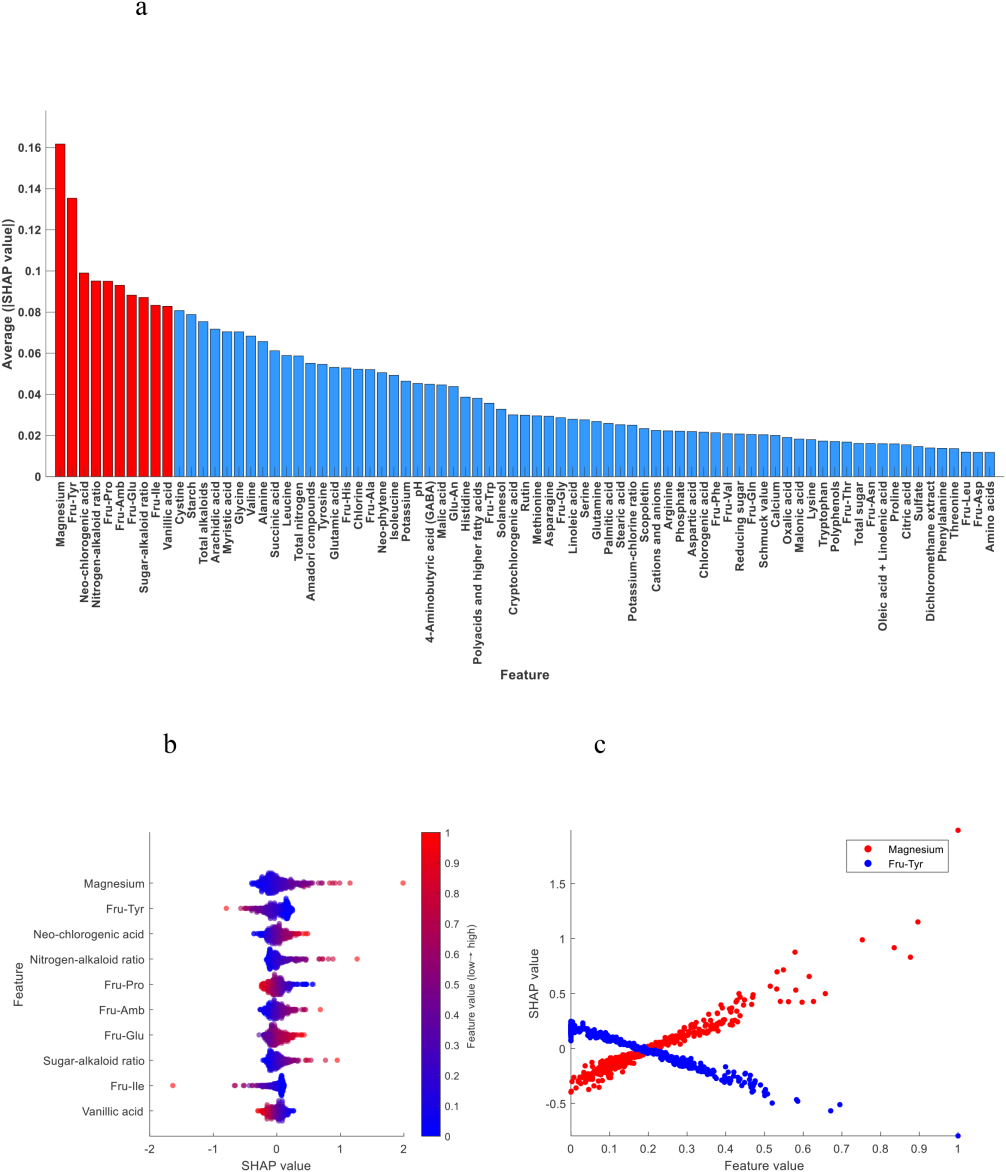
**(a)** Importance ranking of chemical indexes; **(b)** SHAP summary plot of the top 10 chemical indexes; **(c)** SHAP dependence plot of magnesium and Fru-Tyr.

As shown in **Figure 5a**, the top 10 most important chemical indexes in the discrimination model of the lower tobacco leaves to the other positions of tobacco leaves (upper and middle) were magnesium, Fru-Tyr, neo-chlorogenic acid, nitrogen-alkaloid ratio, Fru-Pro, Fru-Amb, Fru-Glu, sugar-alkaloid ratio, Fru-Ile and vanillic acid.

**Figure 5b** shows the SHAP summary plot of the top 10 most important chemical indexes. We found that magnesium, neo-chlorogenic acid, nitrogen-alkaloid ratio, Fru-Amb, Fru-Glu and sugar-alkaloid ratio contributed positively to the model discrimination, that is, with the increase of the content, the model tended to classify tobacco leaves as the lower leaves. Fru-Tyr, Fru-Pro, Fru-Ile and vanillic acid contributed negatively to the model discrimination, that is, with the increase of content, the model tended to classify tobacco leaves as other positions.

**Figure 5c** shows the SHAP dependence plot of magnesium and Fru-Tyr, with the increase of magnesium content, the SHAP value gradually increased, and the model tended to classify tobacco leaves as lower leaves. With the increase of Fru-Tyr content, the SHAP value also gradually decreased, and the model was more tended to classify tobacco leaves as other positions.

## 4 Discussion

Tobacco is an important economic crop, and its tobacco leaf position is closely related to its quality. This study found that the choice of kernel function of SVM will affect the distinguishing effect of tobacco leaf position model, and the accuracy of SVM-hybrid kernel discrimination model was the highest. The possible reason is that the hybrid kernel function combines the characteristics of multiple kernel functions and can capture different levels and types of features in the data. For example, the linear kernel function is suitable for processing simple linear data, the polynomial kernel function is suitable for handling situations with interactions or nonlinear features, the Gaussian kernel function is capable of processing local information, and the sigmoid kernel function is similar to activation function in neural network, which is good at processing continuous nonlinear patterns. The hybrid kernel function can be weighted among these kernels, making the model more flexible and thus improving the ability to classify and generalize complex data. This is also consistent with previous studies (Zhang, 2001). At the same time, it was also found that the discrimination effect using SVM-hybrid kernel was better than that of RF and BPNN. The possible reason is that SVM is more suitable for processing limited samples, while BPNN and RF are suitable for processing large-scale data.

At the same time, it was found that the discrimination rate of lower tobacco leaves was lower than that of upper and middle tobacco leaves, which may be due to the greater influence of light, nutrition and water on lower tobacco leaves, which may lead to their inconsistent characteristics and increase the difficulty of classification. Moreover, due to the longer growth cycle of the lower tobacco leaves, they may be affected by more pests and diseases and the environment, resulting in poor quality and appearance, which leads to ambiguity in the judgment of the model.

According to SHAP algorithm, the top 10 most important chemical indexes were neo-phytene, oxalic acid, total nitrogen, arginine, starch, rutin, potassium, total alkaloids, polyphenols and malic acid in the discrimination model of the upper tobacco leaves to the other positions of tobacco leaves (middle and lower). Except for starch and polyphenols, there were significant differences for the other 8 chemical indexes. The top 10 most important chemical indexes were neo-phytene, oxalic acid, Glu-An, Fru-Tyr, arginine, alanine, potassium, total nitrogen, Fru-Ile and glycine in the discrimination model of the middle tobacco leaves to the other positions of tobacco leaves (upper and lower). Except for neo-phytene and Fru-Ile, there were significant differences for the other 8 chemical indexes. The top 10 most important chemical indexes were magnesium, Fru-Tyr, neo-chlorogenic acid, nitrogen-alkaloid ratio, Fru-Pro, Fru-Amb, Fru-Glu, sugar-alkaloid ratio, Fru-Ile and vanillic acid in the discrimination model of the lower tobacco leaves to the other positions of tobacco leaves (upper and middle). Except for Fru-Tyr, there were significant differences for the other 9 chemical indexes. In the model interpretation of SHAP algorithm, it was further confirmed whether the chemical indexes with significant differences in the independent sample t-test had a high contribution degree in the model, and the chemical indexes that were not significantly detected by the t-test but played an important role in model discrimination were revealed. The possible reason is that the independent sample t-test is mainly used to compare whether there is a significant difference between the mean values of two different groups on a certain feature. It is suitable for small samples and relatively simple hypothesis testing, but it cannot capture the complex interaction relationship and nonlinear mode between features. Many machine learning models (such as SVM, BPNN, RF) are able to capture nonlinear relationships and complex interaction effects that traditional t-test methods cannot achieve. SHAP algorithm can reveal these nonlinear relationships and interaction effects, so that some features that seem unrelated in a single analysis can still play an important role in model discrimination when the model is comprehensively analyzed. Therefore, although the independent sample t-test fails to detect significant differences in the mean values of certain chemical indexes, this does not mean that these characteristics have no effect on the model. Some chemical indexes may not differ significantly in the mean value of each position, but their small changes, when combined with other features, may effectively improve the discriminant ability of the model. Combined with independent sample t-test and SHAP algorithm, the role of chemical indexes on model discrimination was discussed from the perspective of statistics and machine learning. The two methods confirm and complement each other, and enhance the transparency and credibility of the model.

The mean ± standard deviation of the top 10 chemical indexes from the SHAP explanation results —upper vs. other positions (middle and lower), middle vs. other positions (upper and lower), and lower vs. other positions (upper and middle)—are listed respectively (**Tables 13**–**15**). The significant differences in chemical index contents among different tobacco leaf positions are mainly influenced by the tobacco plant’s own metabolism, cultivation techniques, physiological growth of leaves, and field management (Zhang et al., 2022). Taking total nitrogen and rutin as examples, the upper tobacco leaves are located at the top of the plant with sufficient light and active metabolism, leading to strong nitrogen accumulation ability and thus higher total nitrogen content; meanwhile, rutin, as a secondary metabolite, is synthesized and increased under light stimulation, so the rutin content in upper leaves is also higher than that in middle and lower leaves.

**Table 13:** The mean ± standard deviation of the top 10 chemical indexes in the discrimination model between upper tobacco leaves and other leaf positions (middle and lower).

Chemical index	Mean ± standard deviation	
	Upper leaves	Other leaves
Neo-phytene (mg/g)	0.93 ± 0.21	1.00 ± 0.20
Oxalic acid (mg/g)	12.48 ± 2.89	10.80 ± 2.79
Total nitrogen (%)	2.30 ± 0.25	1.83 ± 0.22
Arginine (μg/g)	40.12 ± 10.04	26.49 ± 9.95
Starch (%)	5.15 ± 1.62	4.88 ± 1.72
Rutin (mg/g)	11.10 ± 2.75	9.39 ± 2.53
Potassium (%)	1.95 ± 0.36	2.34 ± 0.49
Total alkaloids (%)	3.10 ± 0.61	1.90 ± 0.56
Polyphenols (mg/g)	25.97 ± 5.69	25.46 ± 4.48
Malic acid (mg/g)	49.20 ± 19.02	59.29 ± 23.59

**Table 14:** The mean ± standard deviation of the top 10 chemical indexes in the discrimination model between middle tobacco leaves and other leaf positions (upper and lower).

Chemical index	Mean ± standard deviation	
	Middle leaves	Other leaves
Neo-phytene (mg/g)	0.97 ± 0.20	0.98 ± 0.22
Oxalic acid (mg/g)	10.42 ± 2.62	12.42 ± 2.91
Glu-An (μg/g)	219.43 ± 47.29	275.27 ± 59.29
Fru-Tyr (μg/g)	68.86 ± 12.79	62.42 ± 13.57
Arginine (μg/g)	25.55 ± 8.43	37.58 ± 11.93
Alanine (μg/g)	509.67 ± 98.84	589.42 ± 102.06
Potassium (%)	2.26 ± 0.42	2.13 ± 0.53
Total nitrogen (%)	1.85 ± 0.23	2.16 ± 0.33
Fru-Ile (μg/g)	18.51 ± 4.58	17.84 ± 3.91
Glycine (μg/g)	33.07 ± 7.18	42.63 ± 9.95

**Table 15:** The mean ± standard deviation of the top 10 chemical indexes in the discrimination model between lower tobacco leaves and other leaf positions (upper and middle).

Chemical index	Mean ± standard deviation	
	Lower leaves	Other leaves
Magnesium (%)	0.48 ± 0.22	0.37 ± 0.15
Fru-Tyr (μg/g)	68.05 ± 12.08	65.25 ± 13.75
Neo-chlorogenic acid (mg/g)	2.05 ± 0.32	1.62 ± 0.36
Nitrogen-alkaloid ratio	1.36 ± 0.43	0.87 ± 0.19
Fru-Pro (μg/g)	6891.82 ± 2403.38	9643.40 ± 2307.56
Fru-Amb (μg/g)	2388.45 ± 454.54	2190.80 ± 381.83
Fru-Glu (μg/g)	854.44 ± 167.05	722.71 ± 153.49
Sugar-alkaloid ratio	21.67 ± 7.91	13.82 ± 6.58
Fru-Ile (μg/g)	20.07 ± 4.59	17.90 ± 4.14
Vanillic acid (mg/g)	0.11 ± 0.01	0.13 ± 0.02

In addition, the mean ± standard deviation of the top 10 chemical indexes in the discrimination models of upper vs. other positions (middle and lower), middle vs. other positions (upper and lower), and lower vs. other positions (upper and middle) leaves were discussed and analyzed together with the SHAP explanation results. In the SHAP analysis, higher the chemical index contents corresponded to higher SHAP values, indicating that the model tended to classify the sample as belonging to the current category — that is, the chemical index made a positive contribution to the discrimination. Conversely, lower the chemical index contents corresponded to higher SHAP values, indicating that the model tended to classify the sample as belonging to the current category — that is, the chemical index made a negative contribution to the discrimination.

The SHAP explanation results were generally consistent with the mean values: chemical indexes that made positive contributions to the discrimination of a given category also showed higher mean values in that category compared to others, while those with negative contributions had lower mean values. Only few chemical indexes were exceptions. The reasons for these discrepancies were analyzed. Feature interaction effects may influence the results, as machine learning models (such as SVM) are not simple linear regressions and often capture nonlinear relationships or interactions between features. Therefore, even if a certain feature has a higher mean value in a specific leaf position, its contribution to the classification outcome may be reversed due to synergistic or antagonistic effects with other features.

At the same time, previous studies mainly investigated the differences in chemical indexes such as total alkaloids, total nitrogen, reducing sugar, potassium, sugar-alkaloid ratio, Amadori compounds, and rutin among different positions of tobacco leaves. For example, Wang et al. (2009) found that in flue-cured tobacco, the total alkaloid content followed the order: upper leaves > middle leaves > lower leaves. The total nitrogen content was highest in the upper leaves, the reducing sugar content was highest in the middle leaves, and the potassium content was highest in the lower leaves. Zhang et al. (2022) found that the reducing sugar content in the middle and lower leaves was significantly higher than in the upper leaves, while the total nitrogen content was significantly lower. The potassium content and sugar-alkaloid ratio were highest in the lower leaves, whereas the total alkaloid content was lowest. Wang et al. (2022) analyzed Amadori compounds across different positions of tobacco leaves and reported the highest levels in the upper leaves, followed by the middle leaves and then the lower leaves. Similarly, Li et al. (2008) observed that rutin content showed the trend: upper leaves > middle leaves > lower leaves.

**Figure 6:**
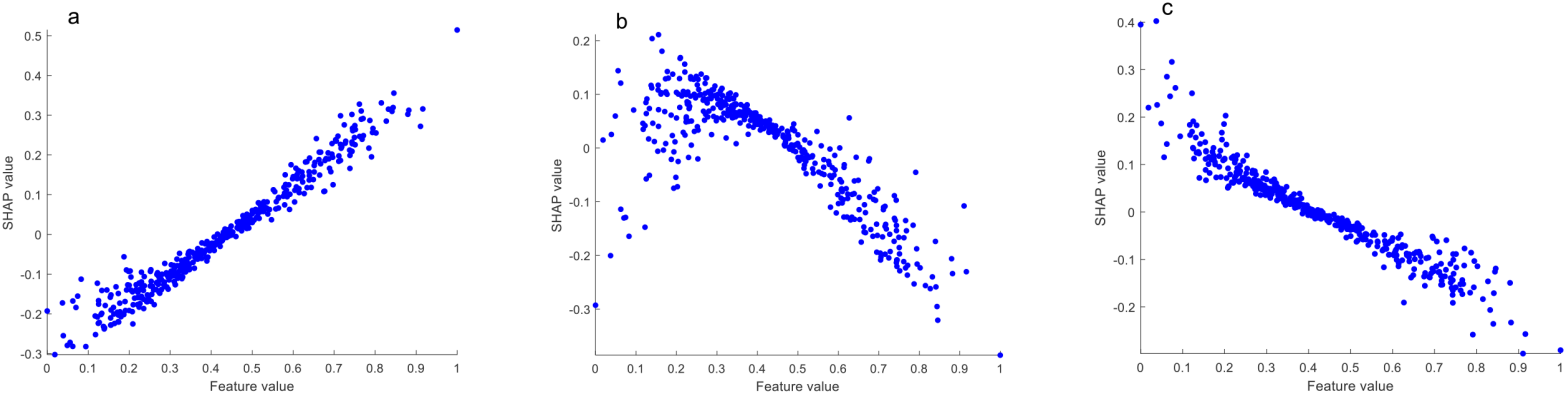
SHAP dependence plots of total alkaloids (**a**, upper leaves vs. other leaves; **b**, middle leaves vs. other leaves; **c**, lower leaves vs. other leaves).

**Figure 7:**
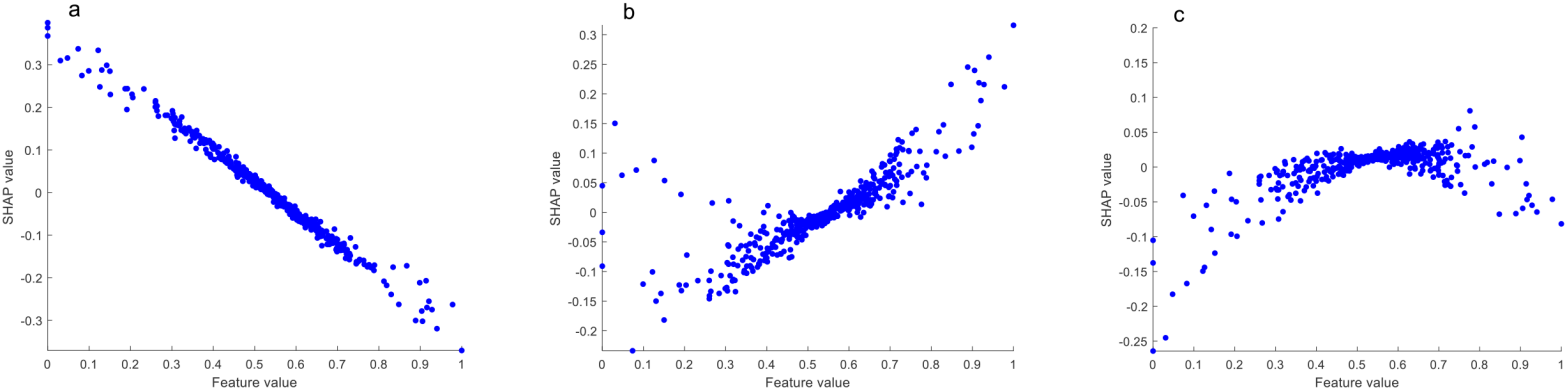
SHAP dependence plots of reducing sugar (**a**, upper leaves vs. other leaves; **b**, middle leaves vs. other leaves; **c**, lower leaves vs. other leaves).

**Figure 8:**
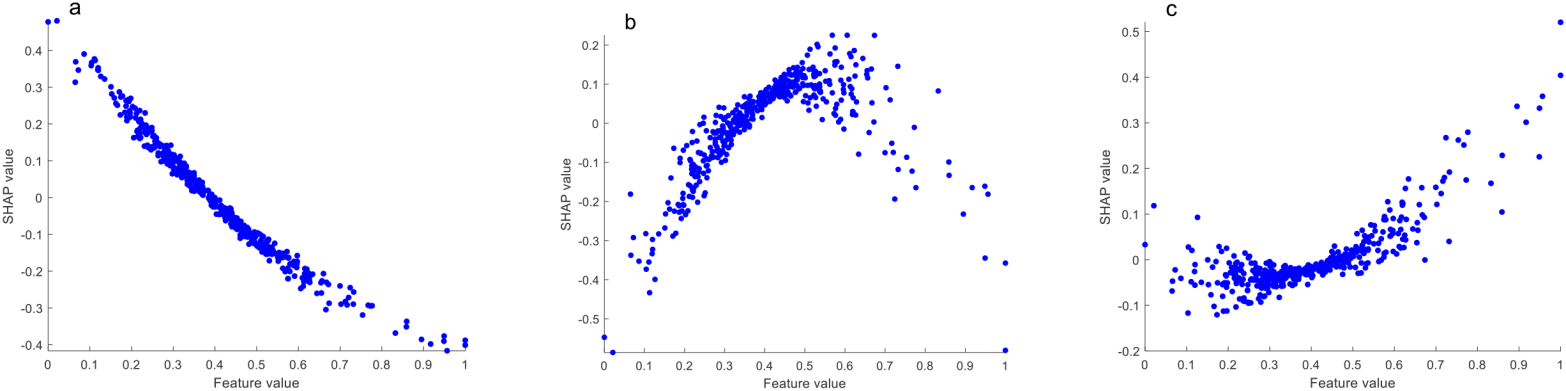
SHAP dependence plots of potassium (**a**, upper leaves vs. other leaves; **b**, middle leaves vs. other leaves; **c**, lower leaves vs. other leaves).

In the SHAP algorithm model interpretation, the higher the total alkaloids content, the more the model tended to classify tobacco leaves as upper leaves. When the content was moderate, the model tended to classify them as middle leaves, and when the content was lower, the model tended to classify them as lower leaves (**Figure 6**). The higher the reducing sugar content, the more the model tended to classify tobacco leaves as middle leaves. When the content was moderate, the model tended to classify them as lower leaves, and when the content was lower, the model tended to classify them as upper leaves (**Figure 7**). The higher the potassium content, the more the model tended to classify tobacco leaves as lower leaves. When the content was moderate, the model tended to classify them as middle leaves, and when the content was lower, the model tended to classify them as upper leaves (**Figure 8**).

The higher the total nitrogen content, the more the model tended to classify tobacco leaves as upper leaves. Conversely, the lower the content, the more the model tended to classify them as leaves from other positions. The higher the sugar-alkaloid ratio, the more the model tended to classify tobacco leaves as lower leaves. Conversely, the lower the ratio, the more the model tended to classify them as leaves from other positions. The higher the Amadori compounds content, the more the model tended to classify tobacco leaves as upper leaves. When the content was moderate, the model tended to classify them as middle leaves, and when the content was lower, the model tended to classify them as lower leaves. The higher the rutin content, the more the model tended to classify tobacco leaves as upper leaves. When the content was moderate, the model tended to classify them as middle leaves, and when the content was lower, the model tended to classify them as lower leaves. The experimental results are consistent with previous studies, demonstrating the reliability of the SHAP algorithm’s model interpretation. (SHAP dependence plots of total nitrogen, sugar-alkaloid ratio, Amadori compounds, and rutin can be found in the **Supplementary File**).

In summary, this study employed a rapid NIR chemical composition analysis technique to obtain 70 chemical components of tobacco leaves. SVM, BPNN, and RF algorithms were used to construct discrimination models for tobacco leaf positions. Furthermore, SHAP algorithm was applied to interpret the impact of each chemical indicator on the model’s predictions. Compared with previous studies, this method enables fast and accurate acquisition of chemical composition, significantly enhances model transparency, and effectively addresses the poor interpretability of near-infrared spectroscopy. This study provides an effective method for crop position traceability and chemical feature analysis.

## 5 Conclusion

The study proposes a novel approach that integrates machine learning with advanced interpretability techniques for both tobacco leaf position discrimination and interpretation. Using the 70 tobacco leaf chemical components obtained using near-infrared chemical component rapid analysis technology, the discrimination model constructed by the SVM-hybrid kernel algorithm optimized by PSO exhibited high accuracy and robustness. The discrimination accuracies reached 98.17% and 96.33% on the training and test sets respectively, which were significantly higher than existing research results. The independent sample t-test revealed significant differences for chemical indexes between leaf positions. The SHAP algorithm was applied to interpret the SVM-hybrid kernel model. The importance rankings of chemical indexes in the discrimination model were provided, and the contributions and specific impacts of each chemical index on the tobacco leaf position discrimination model were analyzed. The SHAP algorithm demonstrated its great potential in explaining tobacco leaf position discrimination models, effectively integrating machine learning with advanced interpretability techniques. This study provides an effective method for crop position traceability and chemical feature analysis.

## Data Availability

The datasets presented in this article are not readily available because permissions are restricted. Requests to access the datasets should be directed to the corresponding authors.

## References

[B1] Anjum M. Khan K. Ahmad W. Ahmad A. Amin M. N. Nafees A. (2022). New SHapley additive ExPlanations (SHAP) approach to evaluate the raw materials interactions of steel-fiber-reinforced concrete. Materials (Basel) 15 (18), 6261. doi: 10.3390/ma15186261, PMID: 36143573 PMC9505950

[B2] Bin J. Ai F.-F. Fan W. Zhou J.-H. Yun Y.-H. Liang Y.-Z. (2016). A modified random forest approach to improve multi-class classification performance of tobacco leaf grades coupled with NIR spectroscopy. RSC Adv. 6, 30353–30361. doi: 10.1039/c5ra25052h

[B3] Cui T. An X. Sun D. Chen D. Zhu Y. (2024). Explainable machine learning models for landslide susceptibility mapping based on SHAP. J. Chengdu Univ. Technol. (Science Technol. Edition) 52(01), 153–172. doi: 10.3969/j.issn.1671-9727.2025.01.11

[B4] Drope J. Siu E. Chaloupka F. J. (2022). Perseverance is innovation: the journey to successful tobacco tax reform. Tobacco Control 31, 241–242. doi: 10.1136/tobaccocontrol-2021-057088, PMID: 35241595 PMC8908804

[B5] Guleria S. Sharma S. K M. S. (2007). Compositional changes in soybean seeds influenced by their positions on stem axis. J. Food Sci. Technol. 44, 607–610.

[B6] Guo J. Zhao L. Liang Y. Wang D. Shang P. Li H.. (2023). Moisture-adaptive corrections of NIR for the rapid simultaneous analysis of 70 chemicals in tobacco: A case study on tobacco. Microchem. J. 189, 108522. doi: 10.1016/j.microc.2023.108522

[B7] He X. Wang P. Chen K. Li Q. Tang J. Yang J.. (2024). Rapid identification of flue-cured tobacco parts based on near infrared spectroscopy and ensemble learning. Chin. J. Anal. Lab. 44 (03), 396–402. doi: 10.3321/j.issn:1004-5708.2008.04.003

[B8] Krizhevsky A. Sutskever I. Hinton G. E. (2017). ImageNet classification with deep convolutional neural networks. Commun. ACM 60, 84–90. doi: 10.1145/3065386

[B9] Li B. Li W. Guo J. Wang H. Wan R. Liu Y.. (2025). Outlier removal with weight penalization and aggregation: A robust variable selection method for enhancing near-infrared spectral analysis performance. Anal. Chem. 97, 7325–7332. doi: 10.1021/acs.analchem.4c07007, PMID: 39970051

[B10] Li L. Yang J. Dai Y. Long J. Li J. Zheng J. (2008). Study on distribution of chlorogenic acid, scopletin,and rutin in flue-cured tobacco. Acta Tabacaria Sin. 04), 13–17.

[B11] Li M. Chen Y. Liu J. Wang Z. Liu X. Zhou X. (2024). Dynamic assessment of multi-customer load adjustable potential of BP neural network based on particle swarm. Power Demand Side Manage. 26, 82–87. doi: 10.3969/j.issn.1009-1831.2024.05.013

[B12] Li S. Han Y. Wang L. Zhang Y. Wang F. Ou Y.. (2025). Machine learning-enhanced flavoromics: Identifying key aroma compounds and predicting sensory quality in sauce-flavor baijiu. Food Chem. 475, 143328. doi: 10.1016/j.foodchem.2025.143328, PMID: 39952173

[B13] Liang Y. Zhao L. Guo J. Wang H. Liu S. Wang L.. (2022). Just-in-time learning-integrated partial least-squares strategy for accurately predicting 71 chemical constituents in Chinese tobacco by near-infrared spectroscopy. ACS Omega 7, 38650–38659. doi: 10.1021/acsomega.2c04139, PMID: 36340111 PMC9631892

[B14] Long T.-Z. Jiang D.-J. Shi S.-H. Deng Y.-C. Wang W.-X. Cao D.-S. (2024). Enhancing multi-species liver microsomal stability prediction through artificial intelligence. J. Chem. Inf. Model. 64, 3222–3236. doi: 10.1021/acs.jcim.4c00159, PMID: 38498003

[B15] Lu Y. Fan X. Zhang Y. Wang Y. Jiang X. (2023). Machine learning models using SHapley additive exPlanation for fire risk assessment mode and effects analysis of stadiums. Sens. (Basel) 23. doi: 10.3390/s23042151, PMID: 36850757 PMC9964004

[B16] Lü X. Wang P. Tang J. Xu J. Li H. Peng Z. (2020). Difference in four derivative indexes of chemical composition in tobacco leaves from different producing areas. Guizhou Agric. Sci. 48, 105–108. doi: 10.3969/j.issn.1001-3601.2020.06.025

[B17] Ni L.-J. Zhang L.-G. Xie J. (2009). Pattern recognition of Chinese flue-cured tobaccos by an improved and simplified K-nearest neighbors classification algorithm on near infrared spectra. Anal. Chimica Acta 633, 43–50. doi: 10.1016/j.aca.2008.11.044, PMID: 19110114

[B18] Rawat W. Wang Z. (2017). Deep convolutional neural networks for image classification: A comprehensive review. Neural Comput. 29, 2352–2449. doi: 10.1162/neco_a_00990, PMID: 28599112

[B19] Richter B. Rurik M. Gurk S. Kohlbacher O. Fischer M. (2019). Food monitoring: Screening of the geographical origin of white asparagus using FT-NIR and machine learning. Food Control 104, 318–325. doi: 10.1016/j.foodcont.2019.04.032

[B20] Rodriguez-Perez R. Bajorath J. (2020). Interpretation of compound activity predictions from complex machine learning models using local approximations and shapley values. J. Med. Chem. 63, 8761–8777. doi: 10.1021/acs.jmedchem.9b01101, PMID: 31512867

[B21] Santos M. R. Guedes A. Sanchez-Gendriz I. (2024). SHapley additive exPlanations (SHAP) for efficient feature selection in rolling bearing fault diagnosis. Mach. Learn. Knowledge Extract. 6, 316–341. doi: 10.3390/make6010016

[B22] Sha Y. Zhao Y. Yu J. Lu T. Liu T. Xie W.. (2019). Difference analysis of six chemical compositions in tobacco parts based on support vector machine. J. Donghua Univ. (Natural Science) 45, 720–723,734. doi: 10.3969/j.issn.1671-0444.2019.05.012

[B23] Theissler A. (2017). Detecting known and unknown faults in automotive systems using ensemble-based anomaly detection. Knowledge-based Syst. 123, 163–173. doi: 10.1016/j.knosys.2017.02.023

[B24] Theissler A. Pérez-Velázquez J. Kettelgerdes M. Elger G. (2021). Predictive maintenance enabled by machine learning: Use cases and challenges in the automotive industry. Reliability Eng. Syst. Saf. 215, 107864. doi: 10.1016/j.ress.2021.107864

[B25] Wang B. Jia G. Zheng W. Chen Y. Liu C. Ai D.. (2022). Correlations between Amadori compound contents and quality of flue-cured tobacco leaves from different ecoregions. Tobacco Sci. Technol. 55, 33–40. doi: 10.16135/j.issn1002-0861.2021.0609

[B26] Wang D. Xie L. Yang S. X. Tian F. (2018a). Support vector machine optimized by genetic algorithm for data analysis of near-infrared spectroscopy sensors. Sens. (Basel) 22 (2), 387–408. doi: 10.3390/s18103222, PMID: 30257420 PMC6210373

[B27] Wang D. Tan D. Liu L. (2018b). Particle swarm optimization algorithm: an overview. Soft Comput.: A Fusion Foundations Methodol. Appl. 18 (10), 3222. doi: 10.1007/s00500-016-2474-6

[B28] Wang D. Yang S. X. (2023). Broad learning system with Takagi–Sugeno fuzzy subsystem for tobacco origin identification based on near infrared spectroscopy. Appl. Soft Comput. 134, 109970. doi: 10.1016/j.asoc.2022.109970

[B29] Wang J. Zhou H. Qin L. Sun S. Zheng C. Xu Z. (2009). The variation analysis of the main chemical components of flue-cured tobacco leaves in Yunnan province. Zhengzhou Univ. Light Industry (Natural Science) 24, 33–37. doi: 10.3969/j.issn.1004-1478.2009.04.010

[B30] Wu T. H. Tung I. C. Hsu H. C. Kuo C. C. Chang J. H. Chen S.. (2020). Quantitative analysis and discrimination of partially fermented teas from different origins using visible/near-infrared spectroscopy coupled with chemometrics. Sens. (Basel) 20 (19), 5451. doi: 10.3390/s20195451, PMID: 32977413 PMC7582835

[B31] Xiang B. Cheng C. Xia J. Tang L. Mu J. Bi Y. (2020). Simultaneous identification of geographical origin and grade of flue-cured tobacco using NIR spectroscopy. Vibrational Spectrosc. 111, 103182. doi: 10.1016/j.vibspec.2020.103182

[B32] Xiao Q. Bai X. Gao P. He Y. (2020). Application of convolutional neural network-based feature extraction and data fusion for geographical origin identification of radix astragali by visible/short-wave near-infrared and near infrared hyperspectral imaging. Sens. (Basel) 20 (17), 4940. doi: 10.3390/s20174940, PMID: 32882807 PMC7506783

[B33] Xie J. Luo J. Yao H. Ni L. Zhang L. (2008). Pattern recognition of growing area and stalk position of domestic flue-cured tobacco based on NIR and chemical components. Tobacco Sci. Technol. 7), 42–44,47. doi: 10.3969/j.issn.1002-0861.2008.07.010

[B34] Xu H. Tian C. Mao R. Gu X. Chang C. (2024). Aerial material consumption prediction model based on PSO-SVM. Modern Inf. Technol. 8, 142–145. doi: 10.19850/j.cnki.2096-4706.2024.08.031

[B35] Yang K. Cai J. Zhang C. Shu R. Liang M. Zhao L.. (2014). Analysis of tobacco site features using near-infrared spectroscopy and projection model. Spectrosc. Spectral Anal. 12), 3277–3280. doi: 10.3964/j.issn.1000-0593(2014)12-3277-04, PMID: 25881423

[B36] Yu C. Ma X. Zhang Y. Li J. Zhao L. Xu L.. (2011). Tobacco plant parts similarity analysis based on near-infrared spectroscopy and SIMCA algorithm. Spectrosc. Spectral Anal. 31, 924–927. doi: 10.3964/j.issn.1000-0593(2011)04-0924-04, PMID: 21714230

[B37] Zhang T. (2001). An introduction to support vector machines and other kernel-based learning methods. AI Magazine: Artif. Intell. 22, 103–104. doi: 10.1609/aimag.v22i2.1566

[B38] Zhang Z. Lin L. Zhang B. Lin H. Chen Q. Chen X.. (2022). Characteristics of main chemical components in different stalk positions of flue-cured tobacco and their relationships with sensory quality. Acta Agric. Jiangxi 34, 28–33. doi: 10.19386/j.cnki.jxnyxb.2022.06.005

[B39] Zhang J. Zong C. (2015). Deep neural networks in machine translation: an overview. IEEE Intelligent Syst. 30, 16–25. doi: 10.1109/MIS.2015.69

[B40] Zhao S. (2022). A study on China’s tobacco taxation and its influencing factor on economic growth. Front. Psychol. 13. doi: 10.3389/fpsyg.2022.832040, PMID: 35282251 PMC8910603

[B41] Zhu Y. Li J. Li J. Li X. Mao L. Yang B.. (2024). Research on optimization of secondary leaf watering parameters by particle swarm optimized random forest algorithm. Softw. Guide 23, 75–81. doi: 10.11907/rjdk.241871

