# Supplementary material for: Construction and interpretation of tobacco leaf position discrimination model based on interpretable machine learning
Source: Front Plant Sci. 2025 Jul 25;16:1619380. doi: 10.3389/fpls.2025.1619380 (PMC12331666; doi:10.3389/fpls.2025.1619380)
Supplement: Supplementary file 1 [file DataSheet1.docx]

**Construction and interpretation of tobacco leaf position discrimination model based on interpretable machine learning**

Ranran Kou^1^,Cong Wang^1^,Jinxia Liu^2^,Ran Wan^1^,Zhe Jin^2^,Le Zhao^1^,Youjie Liu^2^,Junwei Guo^1^,Feng Li^2^,Hongbo Wang^1^,Song Yang^1*^,Cong Nie^1*^

^1^ Zhengzhou Tobacco Research Institute of China National Tobacco Corporation (CNTC), Zhengzhou, China

^2^ Technology Center of China Tobacco Jilin Industrial Co., Ltd., Changchun, China

*** Correspondence:**Corresponding Author:

Song Yang

ztriyangs@163.com

Cong Nie

niec@ztri.com.cn

TABLE S1 The minimum and maximum values of the 70 chemical components in tobacco leaves

| Chemical component | Minimum value | Maximum value |
| --- | --- | --- |
| Total alkaloids(%) | 0.60 | 4.70 |
| Reducing sugar(%) | 10.00 | 35.03 |
| Total sugar(%) | 11.88 | 40.00 |
| Total nitrogen(%) | 1.29 | 3.00 |
| Potassium(%) | 1.00 | 4.00 |
| Chlorine(%) | 0.05 | 2.00 |
| pH | 4.77 | 6.00 |
| Starch(%) | 1.50 | 10.00 |
| Dichloromethane extract(%) | 3.00 | 8.57 |
| Solanesol(mg/g) | 3.00 | 25.00 |
| Sulfate(mg/g) | 2.05 | 30.00 |
| Phosphate(mg/g) | 2.00 | 5.77 |
| Magnesium(%) | 0.16 | 1.27 |
| Calcium(%) | 0.60 | 4.60 |
| Neo-chlorogenic acid(mg/g) | 0.80 | 3.00 |
| Chlorogenic acid(mg/g) | 5.00 | 20.00 |
| Cryptochlorogenic acid(mg/g) | 1.30 | 4.00 |
| Scopoletin(mg/g) | 0.09 | 0.44 |
| Rutin(mg/g) | 4.00 | 17.69 |
| Oxalic acid(mg/g) | 4.00 | 20.00 |
| Malonic acid(mg/g) | 1.17 | 4.00 |
| Succinic acid(mg/g) | 0.15 | 0.42 |
| Malic acid(mg/g) | 10.00 | 120.00 |
| Citric acid(mg/g) | 1.00 | 20.00 |
| Vanillic acid(mg/g) | 0.08 | 0.19 |
| Myristic acid(mg/g) | 0.09 | 0.25 |
| Palmitic acid(mg/g) | 2.15 | 3.60 |
| Linoleic acid(mg/g) | 1.20 | 3.00 |
| Oleic acid + Linolenic acid(mg/g) | 2.58 | 6.00 |
| Stearic acid(mg/g) | 0.40 | 0.80 |
| Arachidic acid(mg/g) | 0.10 | 0.20 |
| Aspartic acid(μg/g) | 100.00 | 825.44 |
| Threonine(μg/g) | 10.00 | 200.00 |
| Serine(μg/g) | 178.72 | 506.43 |
| Asparagine(μg/g) | 150.00 | 5622.16 |
| Glutamic acid(μg/g) | 20.00 | 800.00 |
| Glutamine(μg/g) | 10.00 | 3000.00 |
| Glycine(μg/g) | 15.00 | 80.00 |
| Alanine(μg/g) | 285.31 | 800.00 |
| Valine(μg/g) | 200.00 | 600.00 |
| Cystine(μg/g) | 50.45 | 108.64 |
| Methionine(μg/g) | 6.37 | 20.00 |
| Isoleucine(μg/g) | 3.40 | 19.26 |
| Leucine(μg/g) | 8.11 | 40.00 |
| Tyrosine(μg/g) | 20.00 | 145.58 |
| Phenylalanine(μg/g) | 50.00 | 500.00 |
| 4-Aminobutyric acid (GABA)(μg/g) | 61.02 | 400.00 |
| Lysine(μg/g) | 9.00 | 100.00 |
| Histidine(μg/g) | 30.00 | 400.00 |
| Tryptophan(μg/g) | 30.00 | 400.00 |
| Arginine(μg/g) | 10.00 | 91.44 |
| Proline(μg/g) | 2000.00 | 15000.00 |
| Glu-An(μg/g) | 64.40 | 493.65 |
| Fru-Amb(μg/g) | 1500.00 | 3965.19 |
| Fru-His(μg/g) | 43.19 | 214.67 |
| Fru-Pro(μg/g) | 3000.00 | 15353.22 |
| Fru-Val(μg/g) | 120.00 | 500.00 |
| Fru-Thr(μg/g) | 10.00 | 40.00 |
| Fru-Gly(μg/g) | 15.00 | 36.61 |
| Fru-Ala(μg/g) | 900.00 | 3157.92 |
| Fru-Asn(μg/g) | 1200.00 | 5658.04 |
| Fru-Asp(μg/g) | 500.00 | 1305.99 |
| Fru-Gln(μg/g) | 60.00 | 3816.98 |
| Fru-Glu(μg/g) | 60.00 | 1269.08 |
| Fru-Ile(μg/g) | 15.00 | 49.28 |
| Fru-Leu(μg/g) | 30.00 | 100.16 |
| Fru-Tyr(μg/g) | 50.00 | 139.98 |
| Fru-Phe(μg/g) | 300.00 | 1500.00 |
| Fru-Trp(μg/g) | 50.00 | 900.00 |
| Neo-phytene(mg/g) | 0.40 | 1.40 |

FIGURE S1 SHAP dependence plots of total nitrogen (a: upper leaves vs. other leaves, b: middle leaves vs. other leaves, c: lower leaves vs. other leaves)

FIGURE S2 SHAP dependence plots of sugar-alkaloid ratio (a: upper leaves vs. other leaves, b: middle leaves vs. other leaves, c: lower leaves vs. other leaves)

FIGURE S3 SHAP dependence plots of Amadori compounds (a: upper leaves vs. other leaves, b: middle leaves vs. other leaves, c: lower leaves vs. other leaves)

FIGURE S4 SHAP dependence plots of rutin (a: upper leaves vs. other leaves, b: middle leaves vs. other leaves, c: lower leaves vs. other leaves)
